# Does commercial indoxacarb pose ecotoxicological consequences? Employing a multi-marker approach in the model species *Theba pisana*

**DOI:** 10.1007/s11356-024-33214-z

**Published:** 2024-04-19

**Authors:** Mohamed A. Radwan, Amira F. Gad, Amira M. Abd El-Aziz, Kawther S. El-Gendy

**Affiliations:** 1 Department of Pesticide Chemistry and Technology, Faculty of Agriculture, University of Alexandria, El-Shatby 21545, Alexandria, Egypt; 2 Department of Animal Pests, Plant Protection Research Institute, Agricultural Research Center, Alexandria, Egypt

**Keywords:** Indoxacarb, Land snails, Physiology, Neurotoxicity, Oxidative stress, Histopathology

## Abstract

Indoxacarb is one of the most extensively used oxadiazine insecticides worldwide, but it may exert detrimental effects on ecosystems, population dynamics, and health. Due to the lack of knowledge on the ecotoxicity of indoxacarb, it is still challenging to assess whether this insecticide poses an ecotoxicological impact on terrestrial environments. Therefore, our study aims to provide novel data on the toxic effects of 28-day dietary exposure to commercial grade indoxacarb at two environmentally relevant concentrations, 0.02 µg/mL and tenfold (0.2 µg/mL) on the model species, *Theba pisana*. Their effects were studied using a multiple biomarker approach by evaluating physiological, biochemical, and histopathological responses. After 28 days of treatment, indoxacarb at both concentrations significantly reduced the food intake and growth of the treated snails. Also, it caused decreases in lipid peroxidation (LPO)  levels after 7 and 14 days of exposure, whereas an opposite effect occurred after 21 and 28 days. All treated snails were found to exhibit a lower content of glutathione (GSH) after all times of exposure. Moreover, catalase (CAT), glutathione-S-transferase (GST), and glutathione peroxidase (GPx) activities, as well as protein content (PC), were elevated in the treated snails after all time intervals. Post exposure to both realistic indoxacarb concentrations, changes in acetylcholinesterase (AChE) activity between a decrease and an increase were observed. Furthermore, indoxacarb caused histo-architectural changes in the hepatopancreas of *T. pisana*. Our results demonstrate that, at environmentally relevant concentrations, indoxacarb poses negative consequences for *T. pisana*, indicating its ecotoxicological impacts.

**Graphical Abstract:**

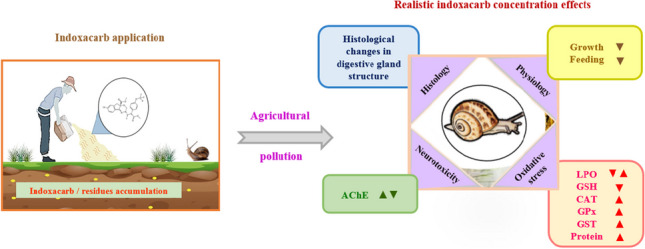

## Introduction

Pesticide pollution associated with anthropogenic activities in the agricultural sector has increased worldwide. Pesticides are a class of hazardous chemicals deliberately applied to the environment to control various pests. Despite the fact that pesticides are vital to modern agriculture to boost agricultural output, their improper use has resulted in a significant environmental risk (Pathak et al. 2022). Consequently, some of these compounds can reach and accumulate in soil ecosystems and then become accessible for assimilation by terrestrial creatures (Sánchez-Bayo 2011). Given the ongoing environmental hazards from pesticide use, it is of utmost importance to assess whether this chemical poses an ecological risk to terrestrial environments. Among these compounds is the insecticide indoxacarb, which may put non-target terrestrial organisms at risk.

Indoxacarb, a promising new generation oxadiazine insecticide, is worldwide used to control sucking and chewing insects (Wing et al. 2000). It exhibits a novel mechanism of action by blocking the sodium channel, causing nervous system depression, paralysis, and death (Lapied et al. 2001). It has a moderate to strong tendency to partition from water to soil and consequently be relatively immobile in soil (U.S. EPA 2000). It is classified as having a moderate risk of bioaccumulation (Lin et al. 2023). The U.S. EPA (2000) reported that indoxacarb degrades well in soil under aerobic conditions (half-life 3 to 693 days). Its environmental profile indicates that considerable amounts of this compound can be found in terrestrial and aquatic environments. It was found that the initial residue of indoxacarb in soil was 0.27 mg/kg (Sdeek and Taha 2018) and in processed persimmons was 0.22 mg/kg (Hulbert et al. 2011). Despite indoxacarb has been shown to be safe for mammals (U.S. EPA 2000), the European Food Safety Authority has recently published a review and update addressing the risks associated with this pesticide to small mammals and bees (EFSA et al. 2018, EFSA 2019). In the soil, indoxacarb residues have exhibited low to moderate risk to arthropods and earthworms (Sakthiselvi et al. 2020). Moreover, it is classified as having a moderate to high risk to freshwater, estuarine/marine fish (Ghelichpour et al. 2019; Ren et al. 2021), aquatic insects (Monteiro et al. 2019), and soil algae (Patra et al. 2022).

Testing a commercial pesticide product, which is actually applied in the environment, is necessary to ascertain the true impact on non-target organisms and the environment because the formulated product’s toxicity is frequently greater than that of the active ingredient alone (Cossia et al. 2020). Excipients and an active ingredient are the two main components of pesticide formulations. Although they are considered inert chemicals due to their lack of pesticide activity, excipients found in commercial formulations can make pesticides more hazardous due to their toxic properties or by promoting the active ingredient’s bioavailability (Rozman et al. 2010). Furthermore, excipients’ impact can be significant to a commercial pesticide’s overall toxicity;  so, it is necessary to consider their effects (Mesnage and Antoniou 2018). It is therefore more realistic to evaluate the toxicity of commercial formulations when examining the impact of pesticides on non-target organisms (Kovačević et al. 2023).

Because chemical monitoring is becoming less informative about ecological effects in ecotoxicological research, the use of biological models (bioindicators) helps directly determine the risk that these residues will actually be present in the terrestrial environment (Van Gestel 2012). Some land snail species are commonly used as bioindicators of soil quality. Based on Gomot de Vaufleury (2000) and Baroudi et al. (2020), land snails have the following characteristics: they are omnipresent and resident organisms, participate in the functioning of the ecosystem and food web, have a well-known ecophysiology, and exhibit remarkable tolerance to environmental pollution. They can also integrate pollution levels and subsequent ecotoxicological effects to gain information on environmental quality. Indeed, as they incorporate the three main routes of exposure, digestive, cutaneous, and pulmonary, land snails are useful tools for assessing risks worldwide (Gomot de Vaufleury and Pihan 2000). The land snail, *Theba pisana*, has the aforementioned characteristics that make it a good model for use as a bioindicator organism (Radwan et al. 2010; Louzon et al. 2023).

Biological markers, or biomarkers, are a sensitive biological technique for assessing pollution-induced stress (Marigomez et al. 2013). They can be evaluated at several biological organization levels (molecules, cells, individuals, and populations), giving them an integrative character of the entire chain of events that follow exposure to pollution (Vasseur and Cossu-Leguille 2003). Pesticides can alter the physiological homeostasis in a variety of taxa, including land snails**,** by inducing oxidative stress via reactive oxygen species (ROS) generation (Radwan et al. 2020; Qiao et al. 2022). Excessive production of ROS in tissues damages the structures of proteins, lipids, and DNA (Cattaneo et al. 2012). The metabolic process by which ROS cause oxidative damage to lipids is known as lipid peroxidation (LPO). This might have a substantial impact on the structure and function of cell membrane and, ultimately, cellular health (Vasilaki and McMillan 2017). To prevent oxidative damage, snails respond to stress by stimulating various antioxidant defense systems such as reduced glutathione (GSH), catalase (CAT), glutathione peroxidase (GPx), and glutathione-S-transferase (GST), which help maintain redox balance (Regoli et al. 2006; El-Gendy et al. 2019a). In addition, acetylcholinesterase (AChE) is an important enzyme involved in nerve signal transmission, and therefore, its alteration constitutes the neurotoxicity of contaminants (Fu et al. 2018). Moreover, histopathology, through the study of histo-architectural alterations in animal tissues demonstrates how pollutant-induced stress affects different tissues and organs (Hamed et al. 2007; Abo-Bakr 2011). In this context, physiological, biochemical, and histopathological markers are useful diagnostic tools and a cost-effective strategy for early-warning detection of environmental pollutants (Sogorb et al. 2014).

To the best of our knowledge, studies on the ecotoxicological consequences of indoxacarb on land snails have not yet been conducted. Therefore, this study aimed to investigate, for the first time, whether indoxacarb-based formulation at two environmentally relevant concentrations, 0.02 µg/mL and tenfold (0.2 µg/mL), poses negative effects on the model species, *T. pisana*. For this study, we selected a multiple biomarker approach by evaluating physiological, biochemical, and histopathological responses. Physiological manifestations were measured by feeding behavior and growth, and biochemical defects were assessed by parameters of oxidative stress: LPO, GSH, CAT, GPx, GST, and a neurotoxic biomarker, AChE, and protein content (PC) after 7, 14, 21, and 28 days of exposure. Histo-architectural alterations in the hepatopancreas of *T. pisana* were also estimated at the end of the experiment.

## Materials and methods

### Chemicals

**Fig. 1:**
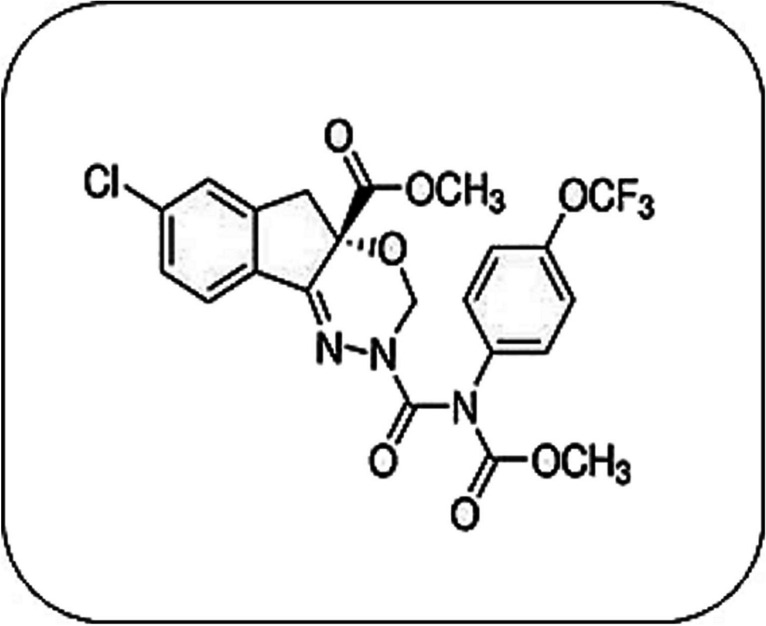
Chemical structure of indoxacarb

The commercial indoxacarb (Truevaunt® 15% SC) used in this study was provided by EgyptChem International for Agrochemicals, Egypt. The chemical structure of indoxacarb is shown in Fig. 1. Thiobarbituric acid (TBA), trichloroacetic acid (TCA), metaphosphoric acid, 5,′5-dithio-bis-(2-nitrobenzoic acid) (DTNB), reduced glutathione (GSH), hydrogen peroxide (H_2_O_2_), cumene hydroperoxide, 1-chloro-2,4 dinitrobenzene (CDNB), acetylthiocholine iodide (ATChI), Folin-Ciocalteus phenol, and bovine serum albumin (BSA) came from Sigma-Aldrich Chemical Co., Germany.

### Tested animals and adaptation

Specimens of 17.3 ± 0.07 mm in shell diameter and 0.94 ± 0.01-g adult terrestrial snail *Theba pisana* were collected in autumn from un-contaminated park in Alexandria , Egypt. In the acclimatization, the gathered snails were kept in ventilated cages (50 × 45 × 45 cm, 200 per cage) for at least 2 weeks prior to the trials in a laboratory setting (27 ± 2 °C, 64 ± 1% RH, and 14:10 L:D photoperiod). Animals were fed leaves of lettuce (*Lactuca sativa*) and starved for 48 h ahead of the experiment. The protocol of the study was approved by Alexandria University’s Animal Ethics Committee (AEC), and experiments were carried out in compliance with the care and handling instructions for animals.

### Diet preparation

To evaluate the toxic effect of indoxacarb on *T. pisana* snails, an artificial food using dry ingredients that included 5-g rabbit meal, 3 g sucrose, and 2 g agar was made based on El-Gendy et al. (2011). In brief, indoxacarb at the requisite concentrations was added to 100 mL of agar medium to combine the dry ingredients. The obtained medium was equally distributed over four Petri dishes. Petri dishes were stored in the refrigerator after cooling*.* During the trials, a stainless-steel cork borer with a 3-cm diameter was used to make diet disks that were provided to the snails. The identical process was used to prepare the control diet, with the exception of distilled water rather than indoxacarb solution.

### Experimental design

To reflect the environmental reality and based on the ecotoxicological profile of indoxacarb (EFSA et al. 2018; Sdeek and Taha 2018), we used two environmentally relevant indoxacarb concentrations: 0.02 and 0.2 µg/mL. Individuals of* T. pisana* were subjected to both concentrations in their diets as described before for 7, 14, 21, and 28 days. In this test, 225 snails (75 individuals/treatment, 3 reps) were separated into 3 categories; the first category acted as the control group and was fed a non-toxic diet*.* The second category of snails received food treated with 0.02 µg/mL indoxacarb while the third category received food treated with 0.2 µg/mL indoxacarb.

### Exposure impact on physiological parameters

#### Feeding and growth responses

Snails of three sets (60 individuals/set, 3 reps) were kept in 12-cm-diameter plastic boxes, where they were given fresh agar disks up to 28 days. For the course of the trial, the boxes were examined every day, water was sprayed on the snails daily to keep them wet, and the diet was replaced every 48 h. Before giving fresh agar disks to the snails, the weight of the disks was measured, and any leftover diet was gathered and dried at 60 °C until it reached a constant weight. It was therefore possible to determine how much dry matter the dried disks actually contained by weighing them, which allowed for the calculation of the snails’ dry matter consumption. The mean weight (g) of dry food ingested per animal per time interval was used to calculate the feeding rate. According to Gomot de Vaufleury (2000), the snail mass was determined by weighing each animal at the beginning of the experiment and again after 7, 14, 21, and 28 days in order to calculate the change in animal mass (g).

### Exposure impact on biochemical parameters

Eighteen snails were chosen at random from each replicate after each time interval and anesthetized using 5% ethanol (d’Ovidio et al. 2019). The snail tissues (hepatopancreas and head-foot) of the selected snails were quickly dissected, cleaned with 0.9% cold saline, weighed, and homogenized with 5 volumes of cold saline for 60s by a Polytron homogenizer (Tekmar tissumizer, Ohio, USA). Using a cooling centrifuge (Janetzki K23 centrifuge, Engelsdorf-Leipzig, Germany), the homogenate was centrifuged at 5000 × g for 20 min at 4 °C. LPO and GSH levels were measured in the homogenate of the hepatopancreas. CAT, GPx, and GST activities, and PC were estimated in the hepatopancreas supernatant, while AChE activity was assessed in the head-foot region.

#### LPO level

Malondialdehyde (MDA) production was quantified at 535 nm using the thiobarbituric acid (TBA) technique established by Placer et al. (1966). Briefly, 0.33 mL of hepatopancreas homogenate was added to 3 mL of freshly prepared TBA reagent consisting of 1:3 volumes of 0.8% TBA and 20% trichloroacetic acid (TCA), respectively. After boiling for 20 min, the mixture was cooled and centrifuged at 3000* g* for 20 min. The MDA level was measured spectrophotometrically using a T-80 + UV/VIS spectrometer PG Instrument Ltd., United Kingdom at 532 nm. The level of LPO is denoted as nmol MDA/g wet tissue.

#### GSH content

Using the method outlined by Owens and Belcher (1965), the content of GSH was measured at 412 nm using 5,′5-dithio-bis-(2-nitrobenzoic acid) (DTNB). The assay mixture contained the homogenate (0.1 mL), 0.5 M phosphate buffer, pH 8.0 (1.5 mL), 3% metaphosphoric acid (0.4 mL), and 0.01 M DTNB (30 µL). After calibration using the standard GSH curve, its content was presented as mg/g wet tissue.

#### CAT activity

The activity of CAT was estimated at 240 nm as illustrated by Beers and Sizer (1952). An aliquot from the hepatopancreas supernatant was mixed with 2 mL of phosphate buffer (66.7 mM), pH 7. The assay was started by adding 1 mL of 12.5 mM hydrogen peroxide (substrate), which was freshly prepared. The time (Δ*t*) needed for a 0.05 decrease in absorbance to be observed is used for the calculation. The activity is represented in unit/g wet tissue.

#### GPx activity

GPx activity was assessed according to Chiu et al. (1976). One-hundred microliters of supernatant was mixed with a mixture containing 100 µL of GSH (1 mM), 100 µL of cumene hydroperoxide (0.05%), and 50 µL of DTNB (0.01 M), and 2.65 mL of Tris–HCl buffer, pH 8.9 (0.4 M). After 1 min, the developed yellow color was measured at 412 nm against a blank (without DTNB). The enzyme activity was displayed as nmol/mg protein.

#### GST activity

The activity of GST was determined as previously described by Vessey and Boyer (1984). The reaction mixture consisted of 10 μL supernatant, 20 µL of 37.5 mM 1-chloro-2,4 dinitrobenzene (CDNB) solution as a substrate, and 0.2 mL of 4 mM GSH solution, and then the volume was completed to 3 mL with 0.1 M phosphate buffer, pH 7.0. The mixture was incubated for 20 min at room temperature, and the absorbance was then measured at 340 nm. The enzyme activity was expressed as μmol/min/mg protein.

#### AChE activity

The activity of AChE was measured based on the Ellman et al. (1961) procedure. The 2.8 mL of 0.1 M phosphate buffer (pH 8), 0.1 mL of head-foot supernatant, and 0.1 mL of DTNB solution made up the reaction mixture. To this mixture, 0.02 mL of freshly prepared acetylthiocholine iodide (ATChI) solution was added. After 10 min, the absorbance was measured at 412 nm. The AChE activity was presented as μmol/min/mg protein.

#### PC

Using bovine serum albumin (BSA) as a standard, protein content was measured in accordance with Lowry et al. (1951). Ten microliters of supernatant was added to 3 mL of medium freshly prepared by mixing 50 mL of reagent A (2 g Na_2_CO_3_ in 100 mL of 0.1 N NaOH) with 1 mL of reagent B (0.5 g CuSO_4_.5H_2_O in 100 mL of a solution containing 1 g sodium potassium tartrate). The mixture was mixed well and incubated for 10 min at room temperature. 0.3 mL of Folin-Ciocalteus phenol solution was added and mixed well. After 30 min, the developed blue color was estimated by recording the intensity at 750 nm.

### Exposure impact on histopathological alterations

At the end of the trial (28 days), the hepatopancreas of three animals from either the control group or those exposed to the tested concentrations of indoxacarb were dissected. The hepatopancreas that had been dissected were preserved for a minimum of 24 h in 10% formalin. The fixative solution was washed overnight under running water. After passing *via* a series of alcohol solutions, the tissue was dried, embedded with wax, cut into 4-μm pieces, mounted on a slide, and dyed with hematoxylin and eosin (Banchroft et al. 1996). The cytological examinations were conducted under 400 × magnification using a Leica DM500 optical microscope, Heerbrugg, Switzerland and photomicrographs were taken in a bright field.

### Data analysis

Data were displayed in all measurements as mean ± standard error (SE). Shapiro–Wilk and Levene’s tests were used to check the results of the parameter tests for normality and homogeneity of variance, respectively. The ANOVA analysis of the data was followed by the separation of the means using the Student–Newman–Keuls test at a probability level (*p* ≤ 0.05). Software Costat V. 2.6 (Costat program 2002) was used for the statistical analysis.

## Results

In the current study, the feeding behavior and growth response of indoxacarb-treated snails were examined as physiological biomarkers to detect any changes that occurred in the animals’ physiology.

### Exposure impact on physiological parameters

#### Feeding behavior

**Fig. 2:**
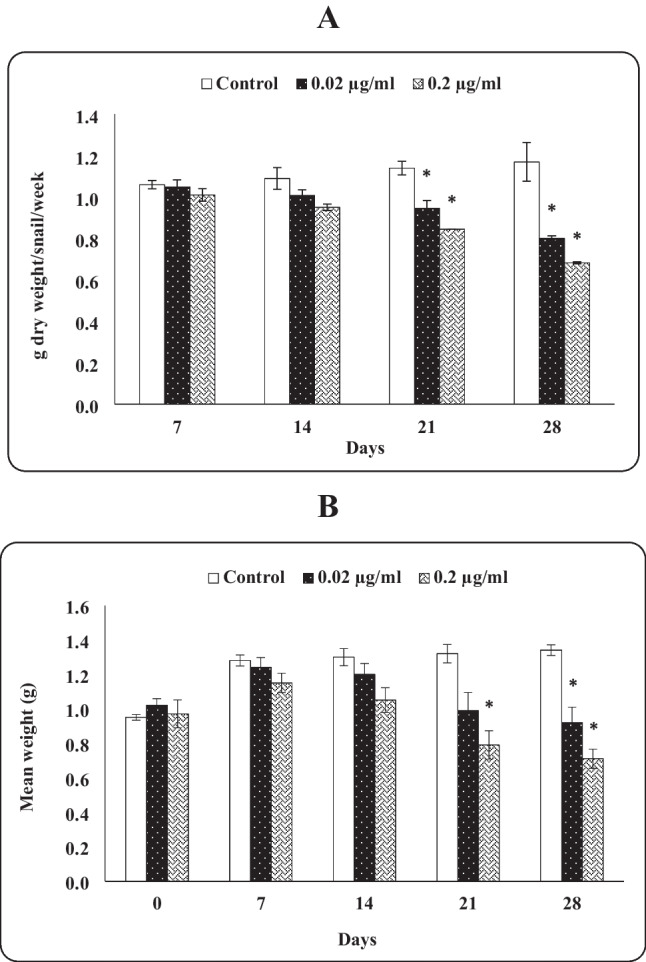
Food consumption (**A**) and growth rate (**B**) of *Theba pisana* snails exposed to contaminated food with the tested concentrations of indoxacarb at various time intervals. Values are mean ± standard error, *n* = 3. *Significantly different from the control value (*p* ≤ 0.05)

The effect of dietary indoxacarb at 0.02 and 0.2 µg/mL on food consumption was noticeable after 7 days of exposure (Fig. 2A). Food intake by snails was significantly decreased after 21 and 28 days of indoxacarb exposure. After 21 days, the control group had ingested 1.14 g dry food/snail, whereas this amount significantly dropped to 0.94 and 0.84 g in the case of snails treated with 0.02 and 0.2 µg/mL of indoxacarb, respectively. Also, the food consumption after 28 days significantly decreased from 1.17-g dry food (control group) to 0.80-g dry food for snails treated with low concentration, whereas it decreased to 0.68-g dry food in snails treated with high concentration of indoxacarb. Moreover, it was observed that the effect of indoxacarb at high concentration on food consumption was more pronounced than at low concentration throughout the experimental exposure periods.

#### Growth response

The mass (represented as mean body weight gain) of control snails was gradually increased throughout the experimental periods. For animals fed on an indoxacarb-contaminated diet, their average mass increased from the beginning of the experiment up to 14 days and then significantly decreased after 21 and 28 days of exposure compared with the control. The mass of snails was 0.99 and 0.92 g when they fed on a 0.02 µg/mL indoxacarb-intoxicated diet and 0.79 and 0.71g on a 0.2 µg/mL indoxacarb-contaminated diet, compared to 1.32 and 1.34 g for the control, after 21 and 28 days, respectively. Overall, the mass of snails subjected to high indoxacarb concentration was less than that of snails exposed to low concentration during 28 days of exposure (Fig. 2B).

### Exposure impact on biochemical parameters

#### LPO level

The obtained results revealed that the LPO level in snails exposed to indoxacarb at the two tested concentrations was non-significantly decrease after 7 and 14 days of exposure. Indoxacarb at 0.02 µg/mL caused an enhancement in LPO level with non-significant differences, however, at 0.2 µg/mL resulted in a significant induction in this respect after 21 days of exposure. Compared to the control sample (1.20 nmol MDA/g.w.t), indoxacarb at low concentration (1.92 nmol MDA/g.w.t) and indoxacarb at high concentration (2.27 nmol MDA/g.w.t) significantly elevated the LPO level after 28-day exposure (Table 1).

**Table 1:** Lipid peroxidation (LPO) level in the hepatopencreas of *Theba pisana* exposed to the two relevant indoxacarb concentrations at different time intervals

Days after exposure	LPO level (nmol of MDA/mg wet tissue)				
	Control	0.02 µg/mL		0.2 µg/mL	
	Mean ± SE	Mean ± SE	% of control	Mean ± SE	% of control
**7**	1.10 ± 0.002^a^	1.03 ± 0.03^a^	93.63	1.06 ± 0.06^a^	96.36
**14**	0.90 ± 0.02^a^	0.87 ± 0.02^a^	96.67	0.89 ± 0.03^a^	98.89
**21**	1.22 ± 0.02^a^	1.27 ± 0.01^a^	104.09	1.50 ± 0.06^b^	122.95
**28**	1.20 ± 0.02^a^	1.92 ± 0.04^b^	160.00	2.27 ± 0.07^c^	189.16

Mean followed by the same letter(s) in each row are not significantly differences from control (*p* ≤ 0.05)

#### GSH content

Data in Table 2 exhibited that both tested concentrations of indoxacarb insignificantly depressed the GSH content of the animal after 7 and 14 days of treatment. Also, it was demonstrated that no significant decrease in GSH content of snails treated with 0.02 µg/mL indoxacarb was observed after 21 days, whereas snails treated with 0.2 µg/mL indoxacarb exhibited a significant decrease in this context. After 28 days, indoxacarb at both tested concentrations caused a significant decrease in GSH content where the mean value of GSH was 5.35 mg/g.w.t in snails exposed to a low concentration and 5.21 mg/g.w.t in snails exposed to a high concentration compared to the control value (5.90 mg/g.w.t).

**Table 2:** Reduced glutathione (GSH) content in the hepatopencreas of *Theba pisana* exposed to the two relevant indoxacarb concentrations at different time intervals

Days after exposure	GSH content (mg GSH/g wet tissue)				
	Control	0.02 µg/mL		0.2 µg/mL	
	Mean ± SE	Mean ± SE	% of control	Mean ± SE	% of control
**7**	6.26 ± 0.33^a^	6.18 ± 0.22^a^	98.72	6.14 ± 0.049^a^	98.02
**14**	6.93 ± 0.03^a^	6.52 ± 0.06^a^	94.08	6.45 ± 0.032^a^	93.11
**21**	6.03 ± 0.14^a^	5.59 ± 0.24^a^	92.72	5.45 ± 0.31^b^	90.44
**28**	5.90 ± 0.03^a^	5.35 ± 0.17^b^	90.57	5.21 ± 0.05^b^	88.19

Mean followed by the same letter(s) in each row are not significantly differences from control (*p* ≤ 0.05)

#### CAT activity

Results clearly indicated that indoxacarb at both concentrations (0.02 and 0.2 µg/mL) significantly enhanced the CAT activity throughout the experimental time in comparison to the control. The activity of CAT in snails exposed to 0.02 µg/mL indoxacarb was elevated by 114.44, 116.23, 142.13, and 131.15% after 7, 14, 21, and 28 days, respectively. Also, the enzyme activity of snails exposed to 0.2 µg/mL indoxacarb was enhanced by 120.78, 122.82, 148.80, and 138.13%, respectively. During the experimental period, indoxacarb at a concentration of 0.2 µg/mL was found to has a greater effect on the CAT activity than at a concentration of 0.02 µg/mL (Table 3).

**Table 3:** Effect of the two realistic indoxacarb concentrations on catalase (CAT) activity of *Theba pisana* after different time intervals of exposure

Days after exposure	CAT activity (units/g tissue)					
	Control	0.02 µg/mL		0.2 µg/mL		
	Mean ± SE	Mean ± SE	% of control		Mean ± SE	% of control
**7**	177.83 ± 6.76^a^	203.51 ± 6.00^b^	114.44		214.79 ± 10.13^c^	120.78
**14**	197.72 ± 4.42^a^	229.81 ± 6.16^b^	116.23		242.85 ± 16.86^c^	122.82
**21**	184.98 ± 0.72^a^	262.92 ± 6.91^b^	142.13		275.26 ± 18.23^c^	148.80
**28**	169.10 ± 10.32^a^	221.78 ± 6.11^b^	131.15		233.58 ± 8.52^b^	138.13

Mean followed by the same letter(s) in each row are not significantly differences from control (*p* ≤ 0.05)

#### GPx activity

The treatment of snails with indoxacarb at 0.02 µg/mL gave a non-significant elevation in GPx activity while the enzyme activity of snails treated with 0.2 µg/mL was significantly increased after 7 days of exposure. Furthermore, indoxacarb at both concentrations caused a significant increase in the activity of GPx after 14 and 21 days of treatment. The mean figures of GP_X_ activity in animals subjected to 0.02 and 0.2 µg/mL indoxacarb were 122.75 and 129.62 nmol/mg protein, respectively, in comparison to the control value (96.35 nmol/mg protein) post14 days. The corresponding values were 170.93 and 178.98 nmol/mg protein, respectively, in comparison to the control value (129.30 nmol/mg protein) post21 days. Both tested indoxacarb concentrations, however, caused a non-significant elevation in the GPx activity of exposed snails after 28 days of exposure (Table 4).

**Table 4:** Effect of the two realistic indoxacarb concentrations on glutathione peroxidase (GPx) activity of *Theba pisana* after different time intervals of exposure

Days after exposure	GPx activity (nmol/mg protein)				
	Control	0.02 µg/mL		0.2 µg/mL	
	Mean ± SE	Mean ± SE	% of control	Mean ± SE	% of control
**7**	95.20 ± 1.42^a^	113.80 ± 4.35^a^	119.53	118.84 ± 1.19^b^	124.83
**14**	96.35 ± 3.40^a^	122.75 ± 0.59^b^	127.40	129.62 ± 2.56^c^	134.53
**21**	129.30 ± 0.81^a^	170.93 ± 6.19^b^	132.19	178.98 ± 1.33^b^	138.42
**28**	112.62 ± 0.80^a^	124.90 ± 3.49^a^	110.90	126.76 ± 12.39^a^	112.55

Mean followed by the same letter(s) in each row are not significantly differences from control (*p* ≤ 0.05)

#### GST activity

As shown in Table 5, a non-significant elevation in the activity of GST in snails subjected to indoxacarb at 0.02 µg/mL; however, a significant induction in the enzyme activity of the snails exposed to 0.2 µg/mL after 7 and 14 days of exposure was observed. After 21 and 28 days, indoxacarb at two tested concentrations induced a significant augmentation in GST activity of snails. The data also showed that the enhancement of GST activity was time- and concentration-dependent.

**Table 5:** Effect of the two realistic indoxacarb concentrations on glutathione-S-transferase (GST) activity of *Theba pisana* after different time intervals of exposure

Days after exposure	GST activity (µmol/min/mg protein)				
	Control	0.02 µg/mL		0.2 µg/mL	
	Mean ± SE	Mean ± SE	% of control	Mean ± SE	% of control
**7**	0.385 ± 0.04^a^	0.392 ± 0.01^a^	101.81	0.435 ± 0.01^b^	112.98
**14**	0.728 ± 0.06^a^	0.802 ± 0.02^ab^	110.16	0.855 ± 0.04^b^	117.44
**21**	0.830 ± 0.07^a^	0.927 ± 0.08^b^	111.68	0.979 ± 0.09^c^	117.95
**28**	0.787 ± 0.04^a^	0.970 ± 0.14^b^	123.25	1.24 ± 0.13^b^	157.96

Mean followed by the same letter(s) in each row are not significantly differences from control (*p* ≤ 0.05)

#### AChE activity

**Fig. 3:**
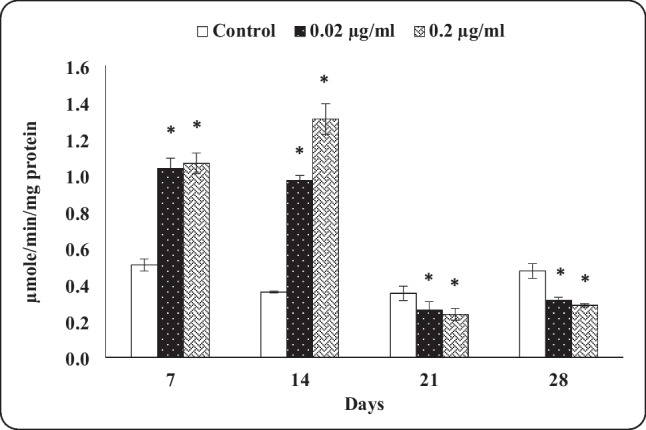
Acetylcholinesterase activity in the head-foot of *Theba pisana* exposed to the two tested concentrations of indoxacarb at different time intervals. Values are mean ± standard error, *n* = 3. *Significantly different from the control value (*p* ≤ 0.05)

The results showed a significant increase in AChE activity for both tested concentrations (0.02 and 0.2 µg/mL) after 7 and 14 days when compared to control snails. However, the opposite trend was observed after 21 and 28 days, where the AChE activity was significantly inhibited. AChE activities of snails exposed to a low concentration of indoxacarb were 205.53, 271.70, 73.71, and 65.96% of control after 7, 14, 21, and 28 days of exposure, respectively. The corresponding values of a high concentration were 209.48, 366.94, 67.42, and 60.04% of control. Generally, a high concentration of indoxacarb was found to be more affected on the AChE activity than a low one (Fig. 3).

#### PC

**Fig. 4:**
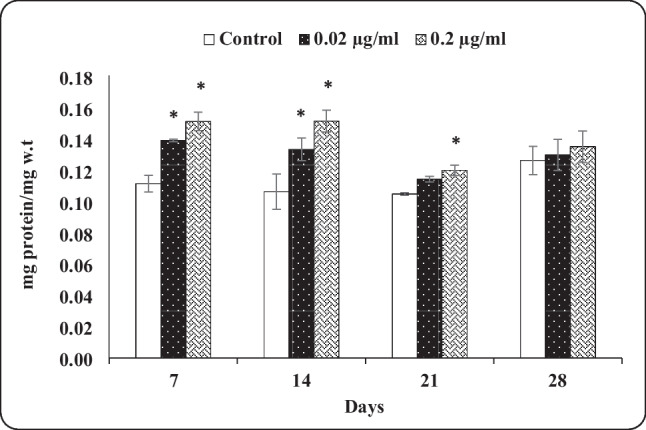
Protein content in the hepatopencreas of *Theba pisana* exposed to the two tested concentrations of indoxacarb at different time intervals. Values are mean ± standard error, *n* = 3. *Significantly different from the control value (*p* ≤ 0.05)

The results clearly indicated that both tested indoxacarb concentrations significantly increased the PC of treated animalsafter 7 and 14 days of exposure. Snails treated with 0.02 µg/mL indoxacarb exhibited a non-significant increase in PC, while the treatment with 0.2 µg/mL indoxacarb gave a significant decrease in this respect after 21 days of exposure. A non-significant elevation in PC was observed in exposed snails to indoxacarb at both tested concentrations after 28 days of exposure (Fig. 4).

### Exposure impact on histopathological alterations

**Fig. 5:**
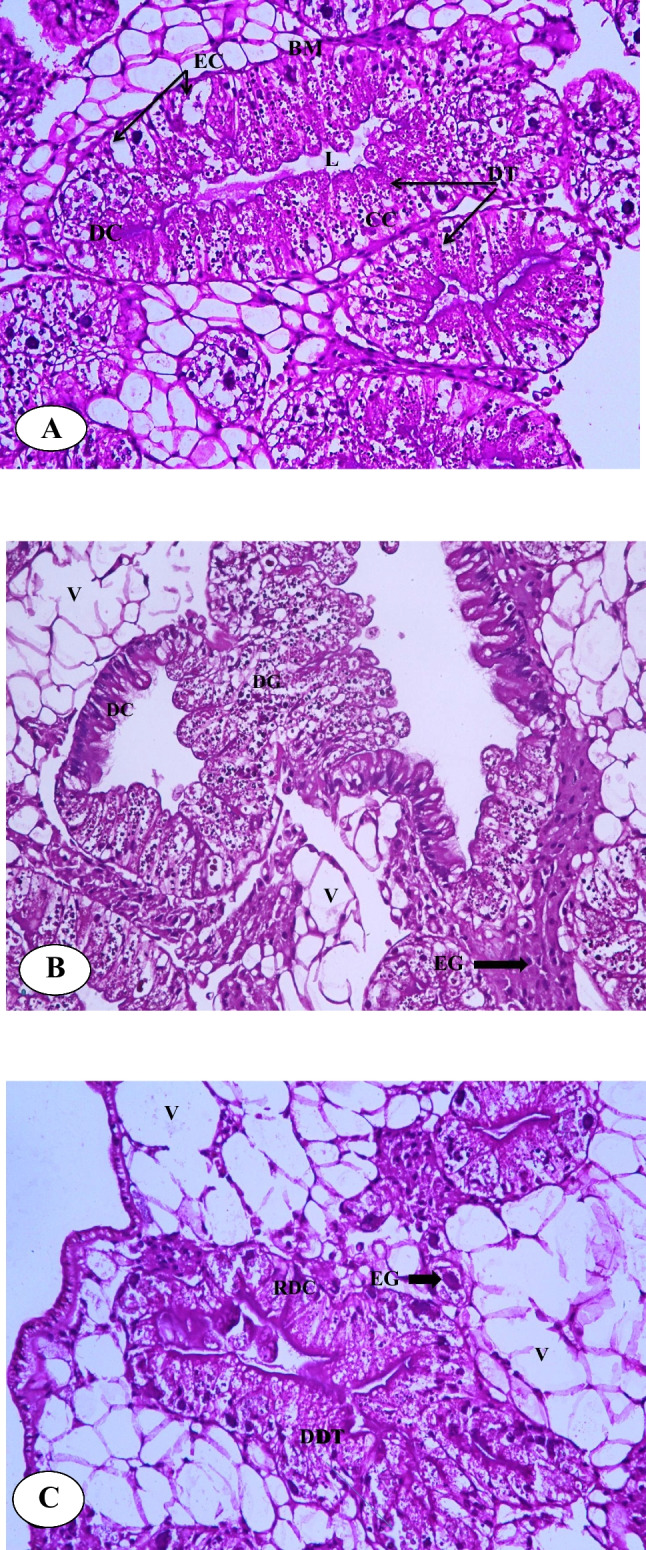
Photomicrograph of the normal hepatopencreas of *Theba pisana* snail (**A**), hepatopencreas of snail treated with 0.02 µg/mL indoxacarb (**B**), and snail treated with 0.2 µg/mL indoxacarb (C) (× 40)

Figure 5A, B, and C show the photomicrographs of control and indoxacarb-treated snail hepatopancreas.

#### Control group

The digestive cells (DC), digestive tubules (DT), excretory cells (EC), lumen (L), calcium cells (C), nucleus (N), and muscle layer (ML) of the un-exposed snails had a normal manifestation, showing no histopathological alterations (Fig. 5A).

#### Experimental groups

In the case of snails subjected to 0.02 µg/mL indoxacarb, the obtained observations displayed various histo-architectural damages, as shown in digestive cells occupied by digestive granules, and the excretory cells were filled with huge vacuoles containing excretory granules of variable size and increased the layer of muscle fiber (Fig. 5B). On the other hand, the group of snails subjected to 0.2 μg/ml indoxacarb exhibited, disruption of digestive tubules, rupture of digestive cells, increase of vacuoles, and the presence of excretory cells with excretory granules of variable size and clumps (Fig. 5C).

## Discussion

The effect of pesticides on environmental compartments has recently become a significant issue on a global scale. Continuous use of pesticides burdens the soil ecosystem, leads to a decline in its health, and may harm soil-dwelling invertebrates that serve as bio-indicators of soil quality. Consequently, more in-depth ecotoxicological data are required for a better grasp of their real risks, as the usage of pesticides is unlikely to decrease in the foreseeable future (Gunstone et al. 2021). Up to now, the eco-toxic effects of indoxacarb on *T. pisana* snails have not been addressed. To the best of our knowledge, this is the pioneer investigation of utmost importance to find out whether indoxacarb-based formulation poses an ecological consequence to the non-target gastropod, *T. pisana*. Therefore, some consequences were evaluated for 28-day exposure to indoxacarb at two environmentally relevant concentrations to elucidate snail physiological, biochemical, and histopathological responses.

In the current study, changes in feeding behavior and growth response of treated snails with indoxacarb were examined as physiological biomarkers. The feeding behavior constitutes one of the earliest reactions to environmental perturbations (McLoughlin et al. 2000), and subsequently, effects on an organisms’ ability to feed may be interpreted into effects on growth, and eventually on populations (Slijkerman et al. 2004). Land gastropods in the wild consume a variety of foods, including dead animal prey and litter, which lead to the accumulation of harmful chemicals in its bodies (Dallinger et al. 2001). Feeding behavior in land gastropods is controlled by the central nervous system, and substantial research is being done to understand how this is accomplished (Chase 2002).

Our findings show that dietary exposure to the two realistic indoxacarb concentrations up to 28 days has been found to reduce the food consumption of snails, indicating physiological stress. The reduced food intake of treated snails might be a result of the indoxacarb neurotoxic effect, which alters their feeding behavior. Substantial reductions in the food intake of snails fed on pollutant-contaminated diets have been reported by many researchers; Gomot de Vaufleury (2000) reported that the food consumption rate of *Canterus aspersus,* subjected to a pentachlorophenol-contaminated diet was sharply decreased. The feeding rate of *C. aspersus* was also significantly reduced after dimethoate exposure (Coeurdassier et al. 2001). Also, significant decreases in the food consumption rate of *T. pisana* were noticed after 2 weeks of exposure to 0.05 LC_50_ of thiamethoxam, abamectin, and acrylamide (El-Gendy et al. 2019b). Furthermore, following 14 days of exposure, *T. pisana* snails consumed significantly less food contaminated by boric acid (Radwan and Gad 2023).

Another potential indictor of physiological integrity is growth measurement (Conti 2008). This index is regarded as a crucial measure of how energetically healthy an organism is under the stress of contamination (Widdows et al. 1997). Various physiological processes can affect growth rate, including alterations in food ingestion, availability, assimilation, and nutrient uptake (Sanders et al. 2018).

In the present study, the growth reduction of the treated snails may be due to the tested compound interference with the feeding activities of these animals. It is possible that decreases in the rate of food consumption may be reflected in decreases of snail total mass.

Our results are consistent with those of Coeurdassier et al. (2001) who established that the growth characteristics of *C. aspersus* decreased after 4 weeks of dimethoate exposure. Moreover, *T. pisana* growth was diminished after dietary exposure to a contaminated diet with thiamethoxam, abamectin, and acrylamide for 14 days (El-Gendy et al. 2019b). Additionally, the growth of *T. pisana* snails was significantly reduced after being fed sub-lethal concentrations of boric acid for 14 days (Radwan and Gad 2023).

It is known that the hepatopancreas is the primary gastropod organ to be impacted by the body’s intake pathway for xenobiotics and is involved in accumulation, detoxification processes, and elimination of pollutants (Dallinger et al. 2001; Radwan et al. 2020). In our study, exposure of *T. pisana* snails to indoxacarb likely induced ROS. This is evidenced by varying stress markers in terms of LPO levels and antioxidant enzyme activities. Therefore, the biochemical and histo-architectural impacts of indoxacarb exposure are predominantly evaluated in the snail hepatopancreas.

LPO is considered a crucial indicator for assessing oxidative stress responses to different xenobiotics. It is a process by which free radicals attack the poly-unsaturated fatty acids of the cellular membrane leading to membrane dysfunction (Halliwell and Gutteridge 2007). The end product of LPO, malondialdehyde (MDA), is employed as a marker of oxidative stress and tissue damage in gastropods (Livingstone et al. 1990). In the current study, the elevation of LPO levels in intoxicated snails may be caused by an excess of ROS or a decrease in the activity of the antioxidant system, suggesting that there is some cellular oxidative injuries as a result of an imbalance in the antioxidative processes (Prased and Muralidhara 2012). On the other hand, the decrease in LPO levels indicated in our investigation can be due to the protective role of the antioxidant system in decreasing LPO levels resulting from ROS. The outcomes of our investigation coincide with preceding findings of Gamil et al. (2011) who revealed a decrease in LPO level after 24 h in second- and fourth-instar larvae of *Spodoptera littoralis* exposed to indoxacarb. In another study, an increase in LPO level was reported in male albino rats after 21-day exposure to indoxacarb (Hassan et al. 2021). Further, Monteiro et al. (2019) reported that exposure of the aquatic midge, *Chironomus riparius*, to 1, 2, 4, and 8 μg/L indoxacarb caused an increase in the level of LPO after 48 h of treatment.

GSH, a low molecular weight tripeptide, plays a crucial role in the detoxifying process. It functions either directly via forming conjugates with electrophilic intermediates or free radicals (Van der Oost et al. 2003) or indirectly via serving as a co-substrate for specific antioxidant enzymes like GST and GPx (Storey 1996). In the current study, the tested compound caused a reduction in the level of GSH in the hepatopancreas of *T. pisana*. This finding could be attributed to radical-induced oxidation and implies an imbalance between antioxidants and oxyradicals that eventually results in oxidative damage (Halliwell and Gutteridge 2007), the pathway of GSH production was negatively affected by xenobiotic stresses (Peña-Llopis et al. 2002), or the consumption of  GSH is associated with an elevation of the enzyme activity of GST (Canesi et al. 1999). Accordingly, our findings completely agreed with the results of Sbartai et al. (2012) who noted that the GSH content decreased in the freshwater ciliated protozoa *Paramecium* sp. from 48 up to 96 h of exposure to indoxacarb. Moreover, a decrease in GSH level in the German cockroach, *Blattella germanica*, treated with LD_50_ and LD_90_ of indoxacarb after 72 h was observed (Maiza et al. 2013).

CAT is an intracellular antioxidant enzyme that catalyzes the conversion of hydrogen peroxide (H_2_O_2_) into O_2_ and H_2_O in order to avoid cellular oxidative damage (Akyilmaz and Dinckaya 2003). In the present study, it was found that CAT activity in *T. pisana* snails was markedly increased, indicating a compensatory elevation in CAT activity to cope with the enhancement of ROS production caused by a pesticide for protecting the cells from oxidative stress conditions (Torres et al. 2002). From the literature survey about activity of the CAT enzyme in organisms exposed to pollutants, contradictory findings have been found. An increase in CAT activity was reported by some authors, while a decrease in its activity was reported by others. *Paramecium* sp., exposed to different concentrations (10, 20, 40, and 80 μm) of indoxacarb, resulted in an increase in CAT activity from 1 to 96 h of treatment (Sbartai et al. 2012). Similarly, indoxacarb enhanced CAT activity in the exposed gammaridean arthropod, *Gammarus kischineffensis* after 48, 72, and 96 h (Demirci et al. 2018). Recently, Ren et al. (2021) found that CAT activity was increased in zebrafish after 28-day exposure to indoxacarb (0.01 mg/L). Indoxacarb exposure decreased the CAT activity of *C. riparius* at concentrations of 2 and 4 μg/L, but increased at the highest tested concentration (8 μg/L) after 48 h (Monteiro et al. 2019).

GPx is an important intracellular antioxidant enzyme for the detoxification of H_2_O_2_ to water to limit its harmful effects (Orbea et al. 2000). Also, GPx stimulates the oxidation of reduced GSH to oxidized glutathione disulfide (GSSG) to facilitate the transformation of peroxides to alcohol (Regoli and Giuliani 2014). In our study, the increased activity of GPx in *T. pisana* snail’s dietary exposed to indoxacarb may be due to the excess of free radical’s production that can stimulate the activity of GPx to scavenge peroxides. Our observations are in harmony with the data published by Monteiro et al. (2019) who showed that an induction in activity of GPx was noticed in *C. riparius* subjected to 1, 2, 4, and 8 μg/L of indoxacarb after 48 h. Likewise, indoxacarb exposure for 20 days caused an increase in the GPx activity of *Ostrinia nubilalis* (Franeta et al. 2018).

GST is a phase II multifunctional enzyme that scavenges ROS in order to preserve the cells against oxidative damage. Additionally, it has a detoxifying role, which can be binding with lipophilic hazardous substances to transform them into hydrophilic substances that are then excreted through metabolic processes (Atamaniuk et al. 2014). In the current research, it was found that the activity of GST in the intoxicated snails increased throughout the exposure periods. The generation of ROS after exposure to the tested compound and the stimulation of the antioxidant defense mechanism by the toxic substance may account for the induction of GST activity (Elia et al. 2007). Our results are corroborated by the work of Monteiro et al. (2019) who reported that indoxacarb increased GST activity in the exposed *C. riparius* after 48 h. An increased GST activity was also observed in experiments with second- and fourth-instar *S. littoralis* larvae exposed to LC_50_ of indoxacarb for 24 h (Gamil et al. 2011). Augmentation in GST was also reported after exposure of* B. germanica* to LD_50_ and LD_90_ of indoxacarb for 72 h (Maiza et al. 2013). Chronic exposure for 20 days to indoxacarb caused a significant enhancement in the GST of *O. nubilalis* (Franeta et al. 2018).

AChE is frequently employed as a reliable neurological endpoint for many environmental contaminants. It plays an essential role in the nervous system *via *hydrolyzing acetylcholine (ACh), which acts as a neurotransmitter (Lionetto et al. 2013). In our investigation, indoxacarb caused an augmentation in the AChE activity at some exposure times, suggesting a negative impact on the snail’s nervous system. The present finding suggests that the tested pesticide increased AChE activity in *T. pisana* snails due to oxidative stress, which was manifested as LPO. In a related study, it was noted that AChE stimulation in the rat brain was associated with a deterioration in the antioxidant status (Carageorgiou et al. 2005). However, it caused suppression of AChE activity in the other exposure intervals, indicating that the tested compound has a neurotoxic action on treated snails. The inhibitory mechanisms may suggest that AChE is unable to hydrolyze acetylcholine . This can lead to the accumulation of ACh, resulting in hyperstimulation, loss of muscular control, and ultimately death (Fulton and Key 2001). AChE activity was also discovered to be particularly sensitive to the effects of free radical (H_2_O_2_) in the 1.6–6.4-μM range, suggesting that a decline in its activity could be related to the impact of a high amount of H_2_O_2_ produced by the LPO pathway (Danylovych 1999). Our results are consistent with earlier research that showed that indoxacarb exposure for 48 h increased the AChE activity of *C. riparius* (Monteiro et al. 2019). In the study of Demirci et al. (2018), AChE activity was found to increase in *G. kischineffensis* subjected to 1/100 LC_50_ indoxacarb after 24-, 48-, 72-, and 96-h exposure. Furthermore, Abdel-Halim et al. (2006) showed that sub-lethal exposure to 0.5 LC_50_ and LC_50_ indoxacarb inhibited the activity of AChE in the soft tissues of the land snail, *Monacha cartusiana*, after 48 h. Inhibition of AChE activity was also observed in *B. germanica* exposed to LD_50_ and LD_90_ indoxacarb for 72-h exposure (Maiza et al. 2013).

Proteins are substantial organic compounds needed by living organisms for tissue construction. They also play a part in cell architecture and energy metabolism. Proteins regulate the interaction process between intracellular and extracellular media (Remia et al. 2008). In the present study, the elevation of PC observed in the intoxicated snails might be the result of a rise in protein synthesis in response to indoxacarb stress. Our results are in accordance with Gamil et al. (2011) who indicated that the PC of *S. littoralis* second-instar larvae treated with indoxacarb increased, whereas the PC of the fourth-instar larvae showed a decrease in comparison to the control.

Our physiological and biochemical data were supported by histopathological observations. Histopathology is a key component of the toxicological and risk assessment of xenobiotics. It is a sensitive, rapid, and valuable marker to demonstrate how xenobiotic-induced sub-lethal stress affects different tissues and organs through the study of histo-architectural alterations in animal tissues, which may also be used to detect environmental hazards from chemicals (Hamed et al. 2007; Radwan et al. 2008). All the histopathological observations in the current investigation indicated that treated snails displayed histo-architectural damage to the hepatopancreas tissues due to indoxacarb stress. Indoxacarb has not been examined for its histopathological impacts on land gastropods, although several injuries to the hepatopancreas of snails caused by several pesticides have been documented by some researchers: Hamed et al. (2007) showed histo-architectural alterations in the hepatopancreas of *Eobania vermiculata* subjected to methiocarb or methomyl using the baiting technique. Both pesticides resulted in significant cytoplasmic vacuolization and disruption with the lowering of microvilli, surface blab formation, an increase in the calcium spherule numbers in calcium cells, and an irregular increase in excretory cell numbers with a lot of excretory granules or residual bodies. After 96 h of exposure, Gaber et al. (2022) revealed that LC_20_ and LC_40_ of methomyl had caused notable degeneration and ruptured digestive cells, some ruptured excretory cells, and the replacement of the cytoplasm in other cells at LC_20_ concentration in *M. cartusiana*.

## Conclusions

The scientific novelty of our research study lies in the fact that for the first time, we evaluated the reactions of the non-target *T. pisana* species to the commercial indoxacarb product by evaluating physiological, biochemical, and histopathological markers to assess the ecotoxicological impact. In this study, it can be concluded that dietary exposure of snails to environmentally relevant concentrations of indoxacarb-based product evokes an adverse impact on animal physiology, oxidative stress and neurotoxicity and caused histo-architectural changes in the hepatopancreas of snails. These tested parameters in snails can be considered useful biomarkers for the diagnosis of indoxacab contamination in terrestrial ecosystems. Since *T. pisana* responds to indoxacarb exposure via biological alterations, it could provide a reliable bioindicator species to monitor the ecological consequences of the compound. Nevertheless, other new and reliable biomarkers, such as omics, need to be identified to provide more risk assessment information on indoxacarb and/or its metabolites in diverse ecosystems. Finally, to minimize the adverse effects arising from indoxacarb exposure, management programs through regulatory bodies should be implemented to prevent ecosystem disruption and the consequences for non-target organisms.

## Data Availability

All data analyzed during this study are included in this article. The raw data that support the findings of this study are available on request from the corresponding author.
